# Precise Editing of chNHE1 Gene via CRISPR/Cas9 Generates ALV-J-Resistant Chicken Primordial Germ Cell

**DOI:** 10.3390/ani15142018

**Published:** 2025-07-09

**Authors:** Xinyi Zhou, Ruyu Liao, Min Tan, Yu Zhang, Haiwei Wang, Keshan Zhang, Qigui Wang, Xi Lan

**Affiliations:** 1 College of Animal Science and Technology, Southwest University, Chongqing 400715, China; a524448729@email.swu.edu.cn (X.Z.); a3029561195@email.swu.edu.cn (R.L.); tanmin123@email.swu.edu.cn (M.T.); zhangyu5789@email.swu.edu.cn (Y.Z.); 2 Chongqing Academy of Animal Sciences, Chongqing 402460, China; wanghw@cqaa.cn (H.W.); zhangks@cqaa.cn (K.Z.); wangqg@cqaa.cn (Q.W.)

**Keywords:** gene editing, CRISPR/Cas9, avian leukosis virus subgroup J (ALV-J), chNHE1, primordial germ cells (PGCs), gene editing

## Abstract

Avian leukosis virus subgroup J (ALV-J), an α-retrovirus, mediates infection by binding to the host-specific receptor chNHE1 (chicken sodium–hydrogen exchanger type 1), leading to immunosuppression and tumorigenesis, which severely threatens the sustainable development of the poultry industry. Studies have shown that the tryptophan residue at position 38 (W38) of the chNHE1 protein is the critical site for ALV-J infection. In this study, we employed the CRISPR/Cas9 system to construct a lentiviral vector targeting the W38 site of chNHE1, transfected it into chicken primordial germ cells (PGCs), and validated its antiviral efficacy through ALV-J infection assays, successfully establishing an in vitro gene-editing system for chicken PGCs. The constructed dual lentiviral vector efficiently targeted the W38 site. PGCs isolated from 5.5- to 7-day-old chicken embryos were suitable for in vitro gene editing. Stable fluorescence expression was observed within 24–72 h post-transfection, confirming high transfection efficiency. ALV-J challenge tests demonstrated that no viral env gene expression was detected in transfected PGCs at 48 h or 72 h post-infection, while high env expression was observed in control groups. After 7 days of infection, p27 antigen ELISA tests were negative in transfected groups but positive in controls, indicating that W38-deleted PGCs exhibited strong resistance to ALV-J. This study successfully generated ALV-J-resistant gene-edited PGCs using CRISPR/Cas9 technology, providing a novel strategy for disease-resistant poultry breeding and advancing avian gene-editing applications.

## Simple Summary

Avian leukosis is a devastating viral disease in the poultry industry, leading to immunosuppression and high mortality in chickens. Scientists have discovered that this virus requires a specific site (W38) on the chicken sodium–hydrogen exchanger 1 (chNHE1) protein—a critical receptor—to facilitate infection. To block viral entry, we employed CRISPR/Cas9-mediated gene editing to precisely delete the W38 site in chicken primordial germ cells (PGCs). Experimental results demonstrated that the edited cells exhibited complete resistance to the virus; no viral genes or proteins were detected post-infection, whereas unedited cells showed massive viral invasion. This study represents the first successful implementation of highly efficient disease-resistant gene editing in chicken PGCs, providing a novel strategy for breeding chickens with innate antiviral immunity. This breakthrough could potentially reduce the poultry industry’s reliance on antibiotics and vaccines, thereby enhancing food safety and poultry health.

## 1. Introduction

Avian leukosis (AL) is a collective term for various neoplastic diseases in poultry caused by the avian leukosis virus (ALV) and the Rous sarcoma virus (RSV) [1]. The primary clinical manifestations include reduced production performance, immunosuppression, and the development of multiple tumors in affected flocks [2]. Since the first reported case of lymphosarcoma by Roloff et al. in 1868, avian leukosis has become globally prevalent, causing significant economic losses to the poultry industry worldwide [3]. At present, due to the lack of effective vaccines and therapeutic drugs, the prevention and control of this disease primarily rely on the detection and culling of positive individuals to implement strict eradication strategies [4,5]. The envelope glycoprotein gp85, encoded by the env gene, serves as the key determinant of viral host specificity and subgroup classification. Based on gp85 amino acid sequences, host range, receptor specificity, and cross-neutralization patterns, ALVs are classified into 11 subgroups (A–K), with subgroups A, B, C, D, E, J, and K being capable of natural infection in chickens [6]. In recent years, subgroup J avian leukosis virus (ALV-J) has emerged as a major global threat to poultry production due to its high pathogenicity and transmissibility, capable of inducing myeloid leukosis and hemangiomas [7].

The infection mechanism of ALV depends on the specific binding between viral envelope proteins and host cell surface receptors [8]. The viral envelope glycoprotein first recognizes and binds to specific receptors on the host cell membrane, followed by fusion between the viral envelope and the cell membrane. During this process, the viral nucleocapsid is released into the cytoplasmic matrix and undergoes uncoating in the cytoplasm. The viral RNA genome is reverse transcribed into complementary DNA (cDNA) by the virus-encoded reverse transcriptase, subsequently forming double-stranded viral DNA. The newly synthesized viral DNA forms a nucleoprotein complex with integrase, which is transported through nuclear pores into the nucleus and integrated into host DNA as a provirus under the mediation of integrase. The integrated proviral DNA utilizes the host cell’s transcription and translation systems to both generate progeny viral RNA genomes and express viral structural and regulatory proteins. These viral components assemble at specific regions of the cell membrane and are released as mature progeny virions through budding. Studies have shown that the receptor for ALV-J infection is chicken sodium–hydrogen exchanger 1 (chNHE1), which regulates the exchange of intracellular H^+^ and extracellular Na^+^, thereby modulating intracellular pH and cell volume [9,10]. The increased activity of NHE1 and the consequent intracellular alkalinization are considered prerequisites for oncogenic transformation and tumor development. The chNHE1 consists of 12 transmembrane domains, six extracellular loops (ECLs), and one intracellular carboxyl terminus [11]. The first extracellular loop (chECL1) of chicken sodium–hydrogen exchanger 1 is the largest extracellular loop of chNHE1 and serves as the critical binding domain for ALV-J and chNHE1 interaction. Four key residues (A30, V33, W38, and E39) in this region have been identified as significantly influencing receptor function [12]. The tryptophan residue at position 38 (W38) serves as the critical amino acid for ALV-J entry, and deletion of W38 significantly enhances host cell resistance to ALV-J infection [13]. Lee et al. employed CRISPR/Cas9 to establish a chNHE1-W38 deletion model in DF-1 cells, followed by ALV-J infection of both mutant and control cells. The results demonstrated that W38 deletion markedly improved the ability of DF-1 cells to resist ALV-J infection, providing an effective target for the development of ALV-J-resistant poultry strains [14]. In 2020, Koslová et al. utilized the CRISPR/Cas9 system to specifically knockout W38 in PGCs, which were then transplanted into sterilized rooster testes to restore spermatogenic function. This approach successfully produced offspring with resistance to ALV-J infection, demonstrating that genetic modification of chicken PGCs enables heritable transmission of viral resistance [15]. Therefore, targeted modification of the W38 site in chNHE1 using CRISPR/Cas9 gene-editing technology can significantly enhance host cell resistance to ALV-J infection, providing a novel strategy for disease-resistant breeding.

As a highly efficient and precise gene-editing tool, the CRISPR/Cas9 system has been extensively applied in genetic modifications of both mammals and poultry [16]. Its core advantage lies in enabling simultaneous multiplex gene editing while substantially shortening the breeding cycle [17]. In avian species, primordial germ cells (PGCs) have become ideal target cells for gene editing due to their unique migratory capacity and germline transmission properties [18]. Recent advancements in feeder-free culture system optimization and cryopreservation techniques have further promoted PGC applications in disease-resistant breeding [19,20].

The inherent complexity of avian reproductive systems, coupled with the unique biological constraints of oviparous development, leads to suboptimal delivery efficiency when employing conventional lipofection techniques or non-viral delivery vectors in avian models. Particularly challenging are the technically demanding procedures required for in vitro isolation and maintenance of chicken primordial germ cells (PGCs) [21]. Furthermore, the design of single-guide RNAs (sgRNAs) targeting avian genomes necessitates careful consideration of multiple parameters, including target sequence specificity and guanine–cytosine (GC) content, to achieve optimal editing efficiency and fidelity. These combined technical hurdles have substantially hindered the widespread implementation of CRISPR-based genome editing technologies in avian species.

Although CRISPR/Cas9 technology has achieved remarkable progress in mammals, its application in poultry disease-resistant breeding remains relatively underdeveloped. Although CRISPR/Cas9 technology has made remarkable progress in mammalian systems, its application in disease-resistant poultry breeding remains relatively underdeveloped. Therefore, combining the unique characteristics of chicken primordial germ cells (PGCs) with CRISPR/Cas9 technology and lentiviral vector systems is expected to demonstrate significant advantages in developing disease-resistant gene-edited chicken breeding. This study aims to employ the CRISPR/Cas9 system for targeted editing of the chNHE1 gene in chicken PGCs, constructing ALV-J-resistant gene-edited PGCs through W38 site knockout, thereby providing novel genetic resources and technical references for poultry disease-resistant breeding. This research will not only contribute to a deeper understanding of ALV-J infection mechanisms but also offer potential gene-editing solutions for avian leukosis prevention and control.

## 2. Materials and Methods

### 2.1. Isolation and Culture of Chicken Primordial Germ Cells (PGCs)

#### 2.1.1. Isolation of Chicken PGCs

Fresh fertilized eggs (obtained from the Chengkou Mountain Chicken Breeding Farm) were placed blunt-end up for one day and incubated at 37.8 °C and 65% humidity for 5.5–7 days (Hamburger–Hamilton stages 28–30). The eggs were then transferred to a constant-temperature chamber. The eggshell surface was wiped with 75% ethanol cotton, and the embryo was extracted from the blunt end and placed in a 100 mm cell culture dish. Under a stereomicroscope, the embryo was positioned with its abdominal cavity facing upward. The abdominal region was incised using a glass microneedle, and the gonads were carefully dissected using sterile forceps. The gonads were rinsed with 1 mL of PBS (containing 1% antibiotic–antimycotic solution) to remove surface blood and then transferred to a centrifuge tube containing 1 mL of PBS. The sample was centrifuged at 1500 rpm for 5 min, and the PBS was aspirated. Next, 1 mL of 0.25% trypsin was added, and the tube was incubated in a 37 °C water bath for 10 min. The tube was vigorously shaken and returned to the water bath for an additional 5 min of digestion. The cell–tissue mixture was transferred to a 5 mL centrifuge tube, and digestion was terminated by adding 1 mL of growth medium. After centrifugation at 1500 rpm for 5 min, the trypsin–medium mixture was removed, and 2 mL of fresh growth medium was added. The cell–tissue suspension was pipetted 30–50 times and filtered through a 40 μm cell strainer into a 12-well plate, followed by incubation at 38 °C with 5% CO_2_ for 4 h.

After 4 h, chicken embryo fibroblast (CEF) cells had mostly adhered. The cell culture supernatant was collected, and the wells were rinsed with 1 mL of growth medium to ensure complete recovery of suspended cells. The collected supernatant was centrifuged at 1500 rpm for 5 min, and the pellet was resuspended in 1 mL of PGCs medium by gentle pipetting (30–50 times). The cell suspension was then transferred to a T25 cell culture flask for further culture, with half-medium changes performed every other day.

Composition of PGC culture medium: KnockOut™ DMEM: 45 mL; B27 supplement (50×): 1 mL; non-essential amino acids (NEAA, 100×): 0.5 mL; β-Mercaptoethanol (55 mM): 0.1 mL; nucleoside mix (100×): 0.5 mL; GlutaMAX™ (200 mM): 0.5 mL; chicken serum: 0.1 mL; recombinant human FGF2 (1 μg/mL): 0.5 mL; activin A (2 μg/mL): 0.5 mL; Sodium pyruvate (100 mM): 0.2 mL; and antibiotic–antimycotic (anti–anti, 100×): 0.5 mL.

#### 2.1.2. Identification of PGCs

(1) RT-PCR Identification

RNA extraction: The PGC suspension from the T25 flask was collected into a 5 mL centrifuge tube and centrifuged at 1500 rpm for 5 min. The supernatant was removed, and total RNA was extracted using the RNAiso Plus reagent (Takara, Japan). Primer design: PGC-specific gene primers were designed based on literature references (Table 1).

RT-PCR: Reverse transcription was performed using the PrimeScript™ RT Reagent Kit with gDNA Eraser (Takara, Japan). The resulting cDNA was amplified by PCR, and the products were separated by 2% agarose gel electrophoresis. The gel was imaged using a gel documentation system.

(2) PAS Staining Identification

PAS staining was performed using the Glycogen PAS staining kit (Solarbio, China). The isolated PGC suspension was dropped onto poly-L-lysine-coated slides, spread evenly, and allowed to adhere for 10 min. Next, 1 mL of PAS fixative was added to completely cover the cells, and the slides were placed in a humid chamber (with a wet towel) for 15 min to ensure complete fixation. The fixative was gently washed off with distilled water, and the slides were air-dried. Then, 1 mL of oxidant solution was applied, and the slides were incubated at room temperature for 15–20 min. After rinsing under running water for 2 min, residual oxidant was thoroughly removed by washing with distilled water (2–3 repeats). Subsequently, 1 mL of Schiff reagent was added, and the slides were stained in a light-protected humid chamber for 10–20 min. The slides were then rinsed with sodium bisulfite solution (2 min, 2–3 repeats), followed by a 5 min wash under running water and additional distilled water rinses (2–3 repeats). Finally, 1 mL of Mayer’s hematoxylin was applied for counterstaining (1–2 min). PGC staining and morphological characteristics were examined under a microscope.

### 2.2. CRISPR/Cas9 Vector Construction

#### 2.2.1. Target Design

The coding sequence (CDS) of the chNHE1 gene was retrieved from the NCBI (National Center for Biotechnology Information). A chNHE1-specific gRNA (5’-CACCGCCCCACGGCTGCTCCCAGGT-3’) was designed using the CHOPCHOP web tool. Additionally, a W38-deletion donor template was constructed: GCCCGCTGCTGCCCGGCCAGCGCTTGCAGGCCGACGCCACGCGGGTCTCCGAGCCCACCGAGCAGCCGTGGGGAGAGCCCGGGGGTATCACCGCCGCCCCGCTGGCCACGGCCCAGGAGGTGCACCCGCTGAACAAACAGCACCACAACCACTC. The designed gRNA and donor template were separately cloned into vectors for lentiviral packaging.

#### 2.2.2. Vector Construction

Construction of LentiCRISPR V2-gRNA1 vector: The LentiCRISPR V2 plasmid was digested using the BsmBI restriction site. The digestion system was gently mixed and incubated at 37 °C in a water bath for 2 h. The gRNA was then cloned into the LentiCRISPR V2 vector. After digestion, DNA gel electrophoresis was performed, and the digested vector was recovered by gel extraction. The gRNA fragment was obtained, with the forward and reverse primers designed as follows: gRNA1-F: CACCGCCCCACGGCTGCTCCCAGGT, and gRNA1-R: AAACACCTGGGAGCAGCCGTGGGGC. The prepared system was gently mixed and placed in a PCR instrument for annealing. Following annealing, DNA ligation was performed. The ligation mixture was incubated overnight at 16 °C in the PCR instrument. The DH5α competent cell suspension, stored at −80 °C, was quickly transferred to an ice bath for slow thawing. After thawing, 50 μL aliquots were dispensed into pre-chilled centrifuge tubes, followed by the addition of approximately 1/10 volume of the ligation product. The mixture was gently mixed and kept on ice for 20–30 min. Subsequently, the mixture was heat-shocked at 42 °C in a constant-temperature water bath for 45 s and immediately returned to the ice bath for 2–3 min. Under sterile conditions, 500 μL of pre-warmed LB liquid medium was added to the transformed cell suspension. The mixture was gently inverted 3–5 times to ensure uniform cell dispersion. The culture was then incubated in a 37 °C constant-temperature shaker at 230 rpm for 45–60 min for recovery. After recovery, the bacterial suspension was evenly spread on LB solid plates containing the appropriate antibiotic. The plates were inverted and cultured in a 37 °C incubator for 12–16 h. The plv3-delW38-copGFP-PURO vector was synthesized by Shenzhen DINGKE Biotechnology Co., Ltd., Shenzhen, China.

#### 2.2.3. Lentiviral Packaging

The LentiCRISPRv2-gRNA and plv3-delW38-copGFP-PURO vectors were co-transfected into 293FT cells (ATCC CRL-11268) using LipoPlus transfection reagent (Sensgene, Nanjing, China). The viral titer was determined by the Reed–Muench method (TCID50 = 10^−3.8^/0.1 mL).

### 2.3. Lentiviral Transfection of PGCs

The PGC cell suspension in a T25 flask was aspirated into a pre-sterilized 5 mL centrifuge tube. Subsequently, 1 mL of PGC medium was added to the T25 flask to gently rinse the bottom, and the medium containing residual PGCs was collected into the same centrifuge tube to ensure complete cell retrieval. The tube was centrifuged at 1200 rpm for 5 min, and the supernatant was discarded. Based on the predetermined optimal MOI, the required volume of lentivirus was transferred into a 5 mL centrifuge tube, diluted to 1 mL with DMEM medium, and supplemented with 2.4 μL of polybrene. The mixture was thoroughly mixed and then added to the PGCs pellet. The cells were gently pipetted 30–50 times to ensure complete resuspension in the lentiviral transfection system, constituting the experimental group. For the control group, 2.4 μL of polybrene was added to PGCs and adjusted to 1 mL with DMEM medium, followed by gentle pipetting (30–50 times) for resuspension. Both the control and experimental groups were prepared in triplicate. The PGCs suspensions from both groups were transferred to a 6-well plate. After 4 h, an additional 1 mL of DMEM medium was added to each well. At 24 h post-transfection, the cell suspensions were collected into 1.5 mL centrifuge tubes, centrifuged at 1200 rpm for 5 min, and the supernatant was removed. The cells were resuspended in 2 mL of PGCs medium, gently pipetted, and transferred back to the 6-well plate for continued culture at 37 °C, 95% humidity, and 5% CO_2_. Fluorescence and cell morphology were observed under an inverted fluorescence microscope at 24 h, 48 h, and 72 h post-transfection.

### 2.4. ALV-J Challenge Assay

Genetically edited PGCs and control PGCs (*n* = 3 replicates per group) were exposed to the ALV-J strain (isolated and preserved in our laboratory). The viral titer was determined by the Reed–Muench method. The viral titer was determined to be 10^3.8^ TCID_50_/0.1 mL. With an MOI of 0.1 and approximately 10^5^ cells per well, the inoculation volume of ALV-J was calculated using the formula V = (MOI × cell count)/viral titer, resulting in approximately 158 μL of viral suspension per well. The final volume was adjusted to 1 mL with DMEM. Viral load was quantified using RT-PCR with ALV-J env-specific primers: forward primer 5’-GCAGCGAGTGACAGGATTGATG-3’ and reverse primer 5’-CACCG-TACAGAACTGGCATTGG-3’. ALV-J p27 antigen was quantified using a commercial ELISA kit (Guosheng Biotech, Wuxi, China).

### 2.5. Statistical Analysis

All data are presented as mean ± SEM from three independent biological replicates. Statistical significance was determined using Student’s *t*-test, with *p* < 0.05 considered statistically significant. The data were statistically analyzed using GraphPad Prism 10.

### 2.6. Cell

Documentation of the 293FT [HEK 293FT] source: purchased from the National Collection of Authenticated Cell Cultures, China; serial: SCSP-5212; identifier: CSTR:19375.09.3101HUMSCSP5212. Documentation of the UMNSAH/DF-1 source: purchased from the National Collection of Authenticated Cell Cultures, China; serial: GNO30; identifier: CSTR:19375.09.3101BIRGNO30. Documentation of the chicken primordial germ cells (PGCs) source: isolated from embryos of Chengkou mountain chickens, sourced from the Chengkou Mountain Chicken Genetic Resources Institute, China; identifier: CSTR:12156.05.1311C0001000000273.

## 3. Results

### 3.1. Vector Construction and Lentiviral Titer Determination

**Figure 1:**
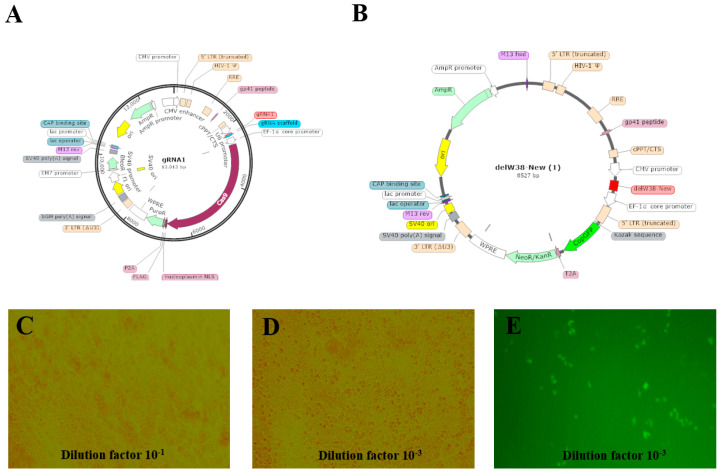
Vector map of W38 site-deletion construct and Lentiviral titer determination results. (**A**) Vector map of LentiCRISPR v2-gRNA; (**B**) Vector map of pLV3-CMV-delW38-EF1α-copGFP-PURO; (**C**,**D**) Lentiviral titer determination results for LentiCRISPR v2-gRNA; (**E**) Lentiviral titer determination results for pLV3-CMV-delW38-EF1α-copGFP-PURO.

The titer of pLV3-delW38-copGFP-PURO virus was 3 × 10^8^ TU/mL. In this study, we successfully constructed a dual lentiviral editing system targeting the W38 site of the chNHE1 gene: the LentiCRISPR V2-gRNA vector contained the specific gRNA sequence, and the pLV3-delW38-copGFP-PURO vector carried the homologous recombination template along with a selection marker. Following packaging in 293FT cells, partial results of lentiviral titer determination are presented in Figure 1. The qPCR analysis revealed that the LentiCRISPR V2-gRNA viral titer reached 2 × 10^8^ TU/mL, and the pLV3-delW38-copGFP-PURO viral titer reached 3 × 10^8^ TU/mL (Appendix A and Appendix B).

### 3.2. Isolation of Chicken Primordial Germ Cells (PGCs)

**Figure 2:**
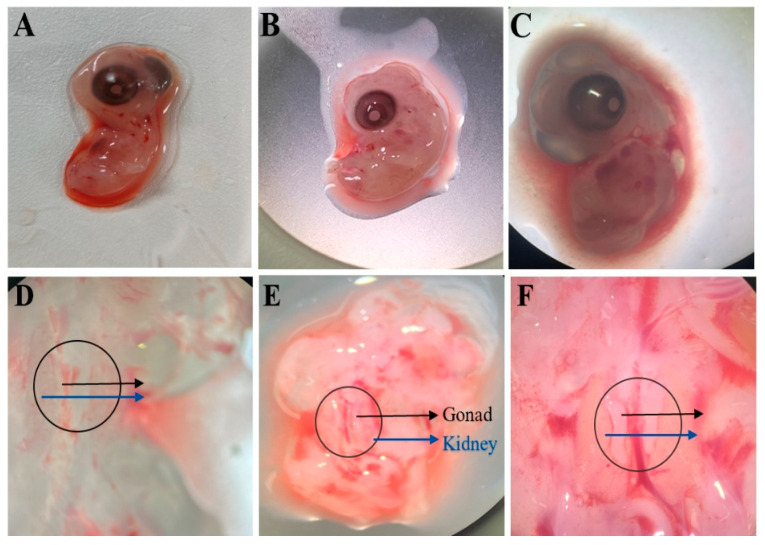
Chicken embryos at 5.5–7 days of age under stereomicroscopy. (**A**,**D**) 5.5-day chicken embryos; (**B**,**E**) 6-day chicken embryos; (**C**,**F**) 7-day chicken embryos.

Under a stereomicroscope, dissections were performed on 5.5-day, 6-day, and 7-day-old chicken embryos. Morphological observations revealed that the embryonic gonads were located bilaterally along the genital ridges adjacent to the mesonephros, presenting as transparent, elongated structures. Notably, the left and right gonads exhibited asymmetric development. In 7-day-old embryos, partial regression of the right gonad was observed, with its size being significantly smaller than the left gonad. Furthermore, the gonads from 7-day-old embryos were more easily isolated due to their larger size (Figure 2).

### 3.3. Purification of PGCs

**Figure 3:**
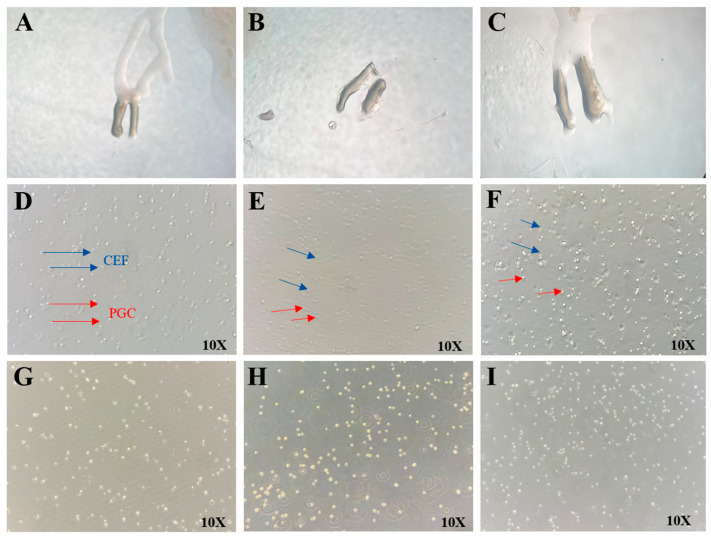
Separation of PGCs. (**A**–**C**) Embryonic gonads; (**D**–**F**) Embryonic gonadal cells; (**G**–**I**) Primordial germ cells (PGCs).

The intact chicken embryonic gonads were successfully isolated (Figure 3A–C). High-purity PGCs were obtained through enzymatic digestion combined with differential adhesion. After 4–6 h of digestion of gonadal cells containing both CEFs and PGCs, the CEFs adhered to the culture surface, displaying a fibroblast-like morphology, while the PGCs remained suspended in the medium (Figure 3D–F). The suspended PGCs were then collected from the cell suspension, with dead cells removed by low-speed centrifugation, and subsequently resuspended in specially formulated PGC culture medium (Figure 3G–I).

### 3.4. Characterization of PGCs

The isolated cells were confirmed as primordial germ cells (PGCs) through three complementary approaches: molecular marker expression analysis, histochemical identification, and genotypic screening.

(1) Molecular Marker Analysis

**Figure 4:**
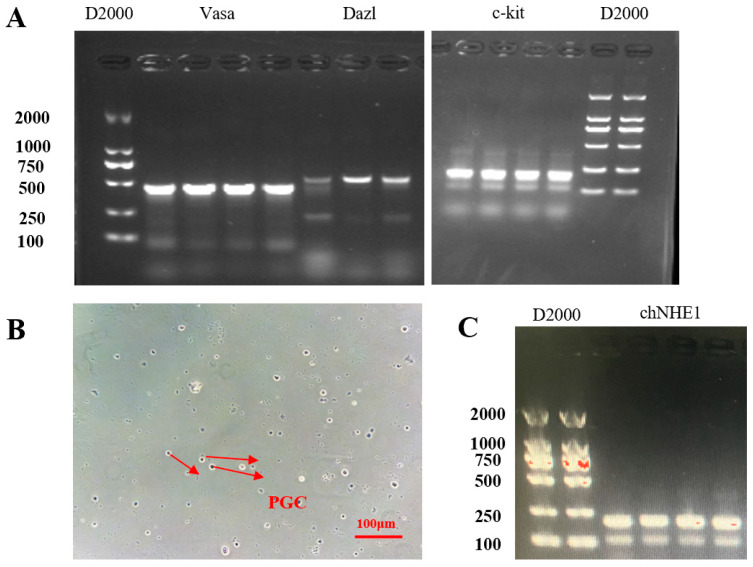
Identification of PGCs. (**A**) RT-PCR analysis of PGC marker genes (Vasa, Dazl, c-kit); (**B**) PAS staining of PGCs; (**C**) RT-PCR detection of chNHE1 gene.

Total RNA was extracted from PGCs, and RT-PCR analysis confirmed the expression of established germ cell markers including Vasa, Daz, and c-kit (Figure 4A).

(2) Histochemical Identification

Periodic acid–Schiff (PAS) staining revealed characteristic features of isolated PGCs: distinctive magenta-red staining pattern, ovoid cellular morphology, and typical diameter range of 15–20 μm (Figure 4B).

(3) Genotypic Analysis

RT-PCR-based screening of the chNHE1 gene in PGC populations demonstrated the absence of natural deletion mutations in all analyzed samples; representative genotyping results are shown in Figure 4C.

### 3.5. Lentiviral Transfection of PGCs

**Figure 5:**
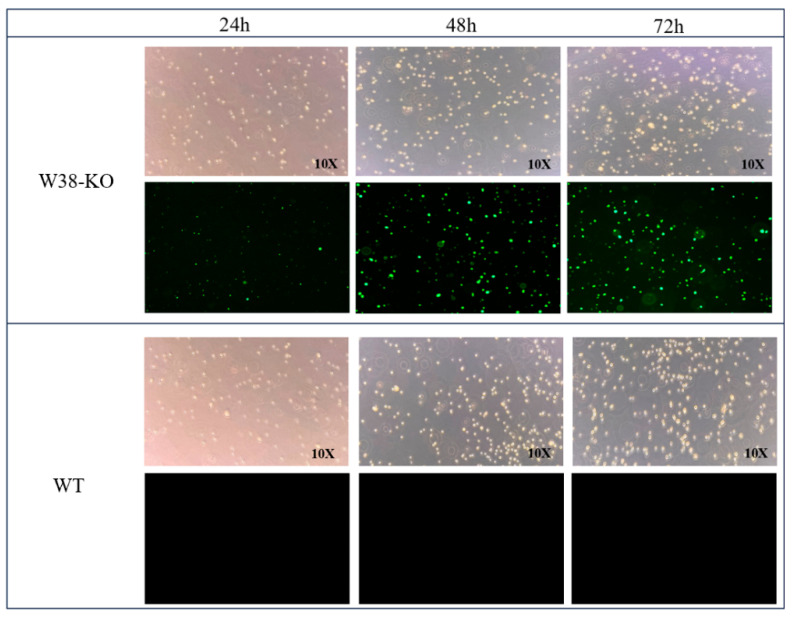
Lentiviral transfection of PGCs.

The W38 knockout lentiviral vector was successfully transfected into PGCs, with fluorescence observation at 24 h, 48 h, and 72 h post-transfection (Figure 5). Significant fluorescence signals were detected at all three time points. The lentivirus-transfected PGCs maintained intact cell membranes and oval morphology while continuing normal proliferation during the 72-h observation period. These genetically modified PGCs were designated as W38 KO-PGCs. These results demonstrate that lentiviral vector transfection represents an efficient method for obtaining gene-edited PGCs with high editing efficiency.

### 3.6. Validation of ALV-J Resistance in W38 KO-PGCs

**Figure 6:**
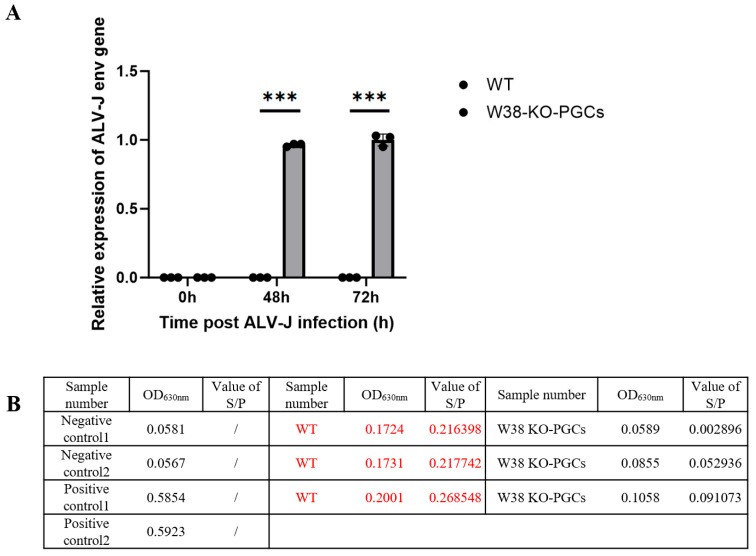
W38-KO-PGCs against ALV-J infection validation. (**A**) Relative expression of env at 48 h and 72 h post-ALV-J infection; (**B**) S/*p* values by ELISA at 7 days post-ALV-J infections not significant (*p* > 0.05); *** *p* < 0.001, *n* = 3; *p* (48 h) < 0.00001; *p* (72 h) < 0.00001.

To evaluate whether lentivirus-transfected PGCs acquired resistance to ALV-J infection, we conducted viral challenge experiments comparing W38 KO-PGCs with control PGCs. Both cell groups were inoculated with the ALV-J strain at 0.1 MOI. As shown in Figure 6A, quantitative analysis of ALV-J env gene expression at 48 h and 72 h post-infection revealed no detectable viral RNA in W38 KO-PGCs, while control PGCs exhibited high levels of env gene expression. Furthermore, cell culture supernatants collected at day 7 post-infection were analyzed using ALV-J p27 antigen ELISA. Figure 6B demonstrates a complete absence of p27 antigen in W38 KO-PGC supernatants, contrasting with strong positive signals in control samples. These findings provide compelling evidence that targeted knockout of a single W38 site in the chNHE1 gene confers complete resistance to ALV-J infection in chicken PGCs.

## 4. Discussion

Avian leukosis virus subgroup J (ALV-J), as a significant pathogen endangering the poultry industry, poses substantial challenges to conventional control measures due to its rapid mutation characteristics. This study developed a novel strategy for breeding ALV-J-resistant chickens by employing CRISPR/Cas9 technology to target the W38 site of chNHE1, a critical ALV-J receptor gene, in chicken primordial germ cells (PGCs). Epidemiological data reveal an alarming 12.7% mutation rate in the ALV-J env gene [22], with novel insertion mutations in the gp85 region potentially leading to diagnostic failures, rendering biosecurity measures alone insufficient for effective outbreak control. Furthermore, traditional eradication strategies through culling result in direct economic losses exceeding 2 million RMB per 10,000 breeding chickens, highlighting the urgent need for developing genetic resistance breeding approaches.

ALV-J infection critically depends on binding to the host cell surface receptor chNHE1, with the W38 site being identified as the essential viral binding domain [12]. Deletion of W38 confers host resistance to ALV-J infection. However, current literature reports no naturally occurring resistance-conferring mutations in chNHE1 among common chicken breeds. This observation prompted our consideration of gene-editing approaches for developing ALV-J-resistant poultry. The CRISPR/Cas9 system, representing third-generation gene-editing technology, offers significant advantages over previous platforms in both design simplicity and editing efficiency, making it the current gold standard for genetic modification. Nevertheless, direct microinjection of gene-editing constructs into chicken embryos demonstrates limited efficiency, and potential cytotoxicity may cause embryonic lethality. Therefore, stem cell-mediated gene editing may represent a more efficient strategy for avian genome engineering.

Primordial germ cells (PGCs) are precursor cells of the avian germline with the potential to differentiate into sperm and oocytes, holding crucial value for poultry gene editing breeding and germplasm conservation [23,24]. PGCs originate from the embryonic epiblast and migrate via the circulatory system to colonize the genital ridges [25].

In vitro gene editing of PGCs followed by transplantation into early-stage chicken embryos enables their circulatory migration to the genital ridges, where they incorporate into gonadal tissues and generate gene-edited offspring [26]. For PGC isolation, enzymatic digestion of gonadal tissues combined with differential plating significantly enhances purification efficiency. Our experiments isolating PGCs from 5.5- to 7-day-old embryos demonstrated no significant difference in PGC proportion within gonadal cell populations across these developmental stages and superior isolation feasibility from 7-day-old embryos due to their larger gonadal size. Thus, 7-day-old embryos represent a more efficient source for PGC extraction. Molecular characterization confirmed PGCs co-express germ cell-specific markers (VASA, DAZL, and c-kit by RT-PCR) and stem cell-related genes. Histochemically, PGCs’ abundant glycogen content produces magenta-red staining in periodic acid–Schiff (PAS) reactions, which we consistently observed in our PAS staining assays. Our PAS staining analysis confirmed that PGCs displayed characteristic ovoid morphology with an average diameter of 15–20 μm. The development of reliable in vitro culture systems for PGCs represents a fundamental requirement for successful genetic modification applications. Early PGC culture methodologies relied heavily on feeder layer systems using CEFs or STO cells, which provided essential extracellular matrices and paracrine signaling factors. However, these traditional approaches suffered from several inherent limitations, including significant batch-to-batch variability, elevated risks of mycoplasma contamination, and technically demanding preparation procedures. In our initial experiments, CEFs isolated from 10-day-old embryos serving as feeder layers demonstrated particular vulnerabilities to microbial contamination, exhibited poor stability during serial passaging, and required labor-intensive maintenance protocols. The field has witnessed significant advancements through the development of serum- and feeder-free culture systems. Research has shown that DMEM/F12-based formulations supplemented with key growth factors, including bFGF, SCF, and BMP4, can satisfy the basic proliferative needs of PGCs [27]. More sophisticated systems using KVa-1 medium supplemented with human bFGF, chicken embryo extract (CEE), and 15% knockout serum replacement have demonstrated the capacity to support stable PGC expansion for over 30 days while maintaining genomic stability and preserving the cells’ native migration capabilities [28]. Building upon these advancements, we have developed an optimized feeder-free culture system that incorporates a serum-free basal medium supplemented with a carefully balanced combination of growth factors, including bFGF2 and activin A. This refined system supports sustained PGC proliferation while maintaining pluripotency markers, enables long-term culture suitable for genetic manipulation, and significantly reduces the contamination risks associated with traditional co-culture approaches. The successful establishment of this robust culture platform provides a reliable foundation for avian germline modification studies.

Currently, the core of avian gene-editing technology lies in utilizing the CRISPR/Cas9 system and its derivative tools to achieve precise genetic modifications in germ cells, combined with in vitro culture and transplantation techniques to establish stably inherited edited lines. Chicken primordial germ cells (PGCs) serve as a reliable gene-editing vehicle, as edited PGCs transplanted into recipient chicken gonads can naturally develop into germ cells and produce genetically modified offspring. Several effective methods exist for PGC gene editing, including lipofection, lentiviral vectors, adenoviral vectors, and electroporation. Compared to adenoviral vectors and lipofection, lentiviral vectors demonstrate higher transfection efficiency and enable stable integration of exogenous genes [29]. Furthermore, lentiviral transfection causes less cellular damage than electroporation. In this study, we employed lentiviral vectors to transfect PGCs, successfully achieving targeted deletion of the W38 site in the chNHE1 gene. The resulting W38-KO-PGCs exhibited resistance to ALV-J infection, demonstrating the effectiveness of this approach. Our findings highlight the potential of lentiviral-mediated PGC editing as a robust platform for generating disease-resistant poultry lines through precise genetic modification.

Although the deletion of W38 significantly enhances the resistance of cells to ALV-J infection, whether the gene function of chNHE1, an important protein for maintaining intracellular pH and ion balance, is affected by W38 deletion still needs to be further studied. In addition, Wang et al. showed that annexin A2 (chANXA2) and chicken glucose-regulating protein 78 (chGRP78) are novel receptors for ALV-J infection hosts, suggesting that they may be new targets for anti-ALV-J virus infection in addition to the chNHE1 gene, but more in vitro and in vivo experiments are needed for validation [30]. The presence of novel receptors suggests the need to develop a multi-target editing strategy in order to achieve broad-spectrum resistance. The advantage of CRISPR/Cas9 lies in its capability to simultaneously edit multiple genes within cells. Although individual editing of chNHE1-W38 has demonstrated significant resistance, combined targeting of chANXA2 or chGRP78 may potentially enhance antiviral effects by blocking additional viral entry pathways. Subsequent studies could employ multiplex sgRNA systems to evaluate synergistic effects, while special attention should be paid to avoiding the accumulation of off-target effects.

The edited PGCs were transplanted into chicken embryos at 2–3 days of development (Hamburger–Hamilton stage 15–16), enabling them to migrate via blood circulation and colonize the genital ridge, where they subsequently differentiated into functional gonadal tissues and ultimately produced gene-edited offspring. However, this in vivo transplantation approach faces significant challenges, including immune rejection and competition with endogenous PGCs [31].

The chNHE1 protein is a critical transporter responsible for maintaining cellular pH and volume, playing a central role in development, metabolism, and stress responses. Its function is highly conserved with mammalian NHE1, making it an important model gene for studying acid-base balance and related diseases. To date, no studies have reported the functional consequences of W38 deletion in chNHE1. In our current study, single-site knockout of W38 did not compromise cellular morphology or proliferation. However, the potential impact of this deletion on chNHE1’s physiological functions requires further investigation.

While the ALV-J-resistant PGCs we obtained hold promise as breeding material for producing ALV-J-resistant gene-edited chickens, the generation of gene-edited individuals must strictly adhere to ethical guidelines and national regulations. Currently, in China, all gene-edited organisms are confined to laboratory research and prohibited from entering commercial markets. Internationally, regulatory policies for gene-edited animals vary significantly across jurisdictions; for instance, the European Union and Japan strictly classify them as GMOs, whereas the U.S. Food and Drug Administration has officially approved four genetically engineered animal products for human consumption. These regulatory disparities not only hinder the widespread adoption of gene-editing technologies in animal husbandry but may also potentially trigger international trade disputes. Consequently, establishing a harmonized international regulatory framework has become one of the most critical tasks for advancing gene-edited agricultural animals.

In this study, a PGC-based ALV-J resistance gene-editing breeding system was successfully established, in which the W38 locus of chNHE1 was accurately deleted, and the obtained W38-KO-PGCs maintained normal morphology, while completely blocking ALV-J infection, and the obtained gene-edited PGCs were used as breeding materials for the production of ALV-J resistant chickens. This study not only provides a new strategy to solve the problem of avian leukemia industry, but also promotes the application and development of avian gene-editing technology.

## 5. Conclusions

This study successfully developed a novel PGC-mediated breeding system for ALV-J resistance through targeted genome editing. The engineered W38-deleted PGCs demonstrated complete resistance to ALV-J infection, representing a significant breakthrough in avian disease-resistant breeding.

## Figures and Tables

**Table 1:** Primers for PCR.

Primer Name	Primer Sequence
*chNHE1*	F: CTGAACAAACAGCACCACAAC
	R: GAGGAGGAAGAGGAAGAAGATG
*Dazl*	F: CGTCAACAAC-CTGCCAAGGA
	R: TTCTTT-GCTCCCCAGGAACC
*Vasa*	F: CACAGCCACACAGAAGACGG
	R: CCATCAAGTCCACAACACGG
*c-kit*	F: GTGGGCAA-GAAGTGGAAGCC
	R: GCAAACCAA-GCATCTCATCCC

**Figure A1:**
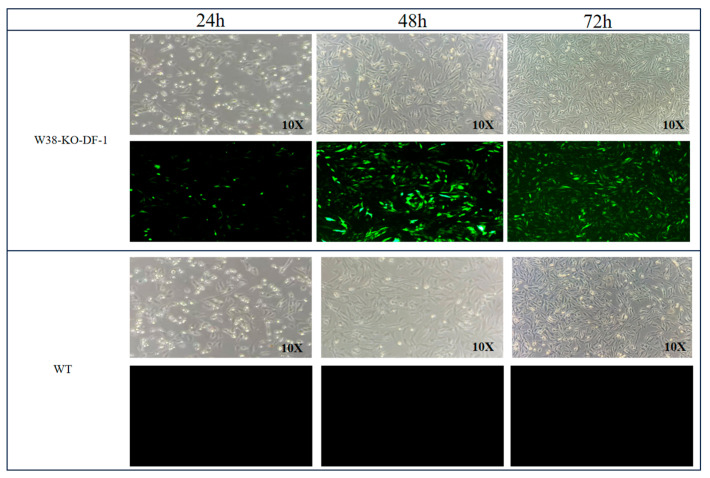
Lentiviral transfection of DF-1.

**Figure A2:**
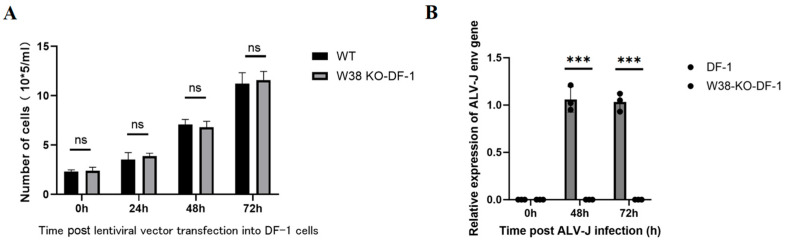
Validation of the effect of W38 deletion on the proliferation level of DF-1 cells and ALV-J infection. (**A**) Number of cells within 72 h of lentiviral transfection; (**B**) Relative expression of env after 48 h and 72 h of ALV-J infection. *** *p* < 0.001, ns: no significance.

**Figure A3:**
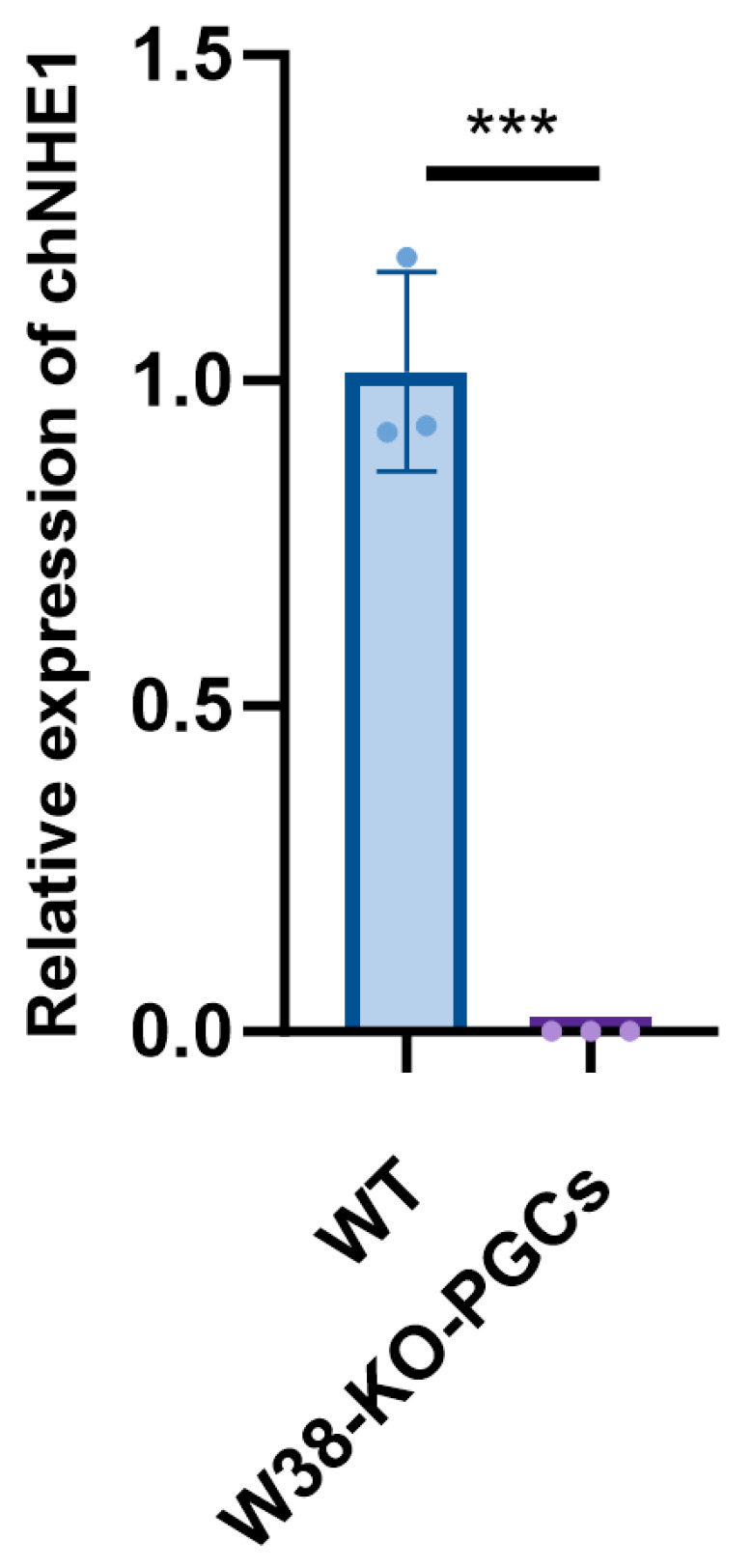
Relative expression of the chNHE1 gene in transfected and control PGCs identified by qRT-PCR. *** *p* < 0.001.

**Figure A4:**
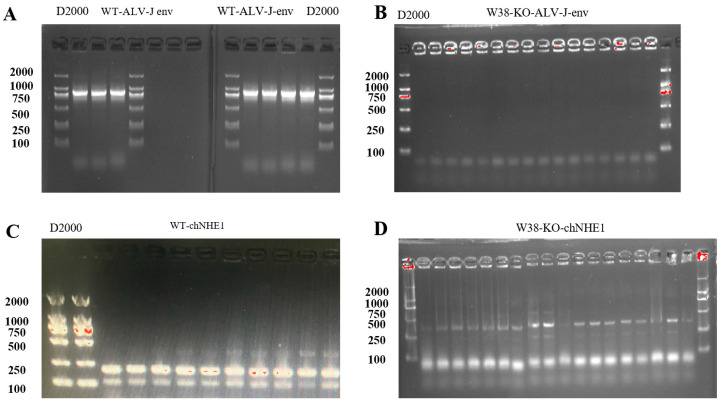
PCR identification of ALV-J env gene and chNHE1 gene in transfected and control PGCs. (**A**,**B**) PCR identification of ALV-J env gene in transfected and control PGCs; (**C**,**D**) PCR identification of chNHE1 in transfected and control PGCs.

**Figure A5:**
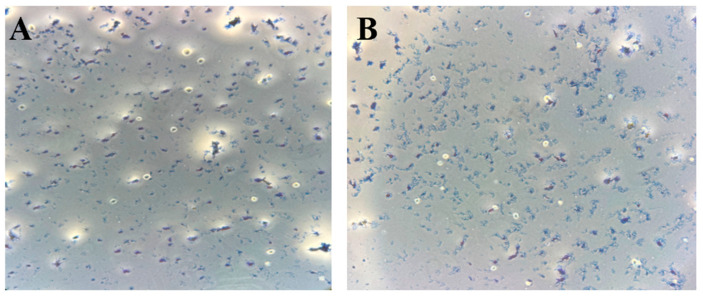
Determination of PGCs activity in transfected and control groups by Formosan blue staining. (**A**) Determination of WT-PGCs activity by Formosan blue staining, (**B**) Determination of W38-KO-PGCs activity by Formosan blue staining.

## Data Availability

The original contributions presented in this study are included in the article material. Further inquiries can be directed to the corresponding author(s).

## Abbreviations

The following abbreviations are used in this manuscript:

ALV-J	Avian leukosis virus subgroup J
chNHE1	Chicken sodium–hydrogen exchanger type 1
PGCs	Primordial germ cells
CRISPR	Clustered regularly interspaced short palindromic repeats
Cas9	CRISPR-associated protein 9
RT-PCR	Reverse transcription polymerase chain reaction
PAS	Periodic acid–Schiff
ELISA	Enzyme-linked immunosorbent assay
MOI	Multiplicity of infection
TCID50	50% tissue culture infectious dose

## Appendix A

The constructed lentiviral transfection system was transfected into DF-1 cells, with blank controls set up. Morphological and fluorescence observations were performed on both transfected and control groups at 24 h, 48 h, and 72 h post-transfection. At 24 h, 48 h, and 72 h after lentiviral transfection, the transfected DF-1 cells exhibited typical elongated fibroblast morphology, consistent with the control DF-1 cells. Significant fluorescence was observed in transfected DF-1 cells at all three time points (24 h, 48 h, and 72 h). No significant difference in cell numbers was found between lentiviral-transfected DF-1 cells and control groups at 24 h, 48 h, or 72 h. Notably, W38 KO-DF-1 cells showed no detectable viral env gene expression at 72 h post-ALV-J inoculation, while high expression of viral env gene was observed in control groups at both 48 h and 72 h after inoculation.

## Appendix B

We evaluated the editing efficiency of our constructed lentiviral system in PGCs through fluorescence observation, qRT-PCR, and PCR validation. Fluorescence observation results are shown in Figure 5, where transfected PGCs exhibited strong green fluorescence within 72 h, while no fluorescent signal was detected in the control group. We have supplemented the qRT-PCR and PCR validation results in Appendix B. Quantitative fluorescence detection of transfected and control PGCs (Figure A3) showed no chNHE1 gene expression in transfected PGCs, whereas high expression was observed in controls. PCR identification results (Figure A4) demonstrated bright chNHE1 gene bands in control PGCs, while transfected PGCs showed either no signal or weak signals for chNHE1.
